# Isolated Cerebral Myeloid Sarcoma in an Allogeneic Stem Cell Transplant Recipient

**DOI:** 10.1002/ajh.27640

**Published:** 2025-02-15

**Authors:** Gabriele Magliano, Silvia Zanon, Rachele de Domenico, Marco Galli, Enrico Morello, Luisa Lorenzi, Gaetano Paolino, Marco Fontanella, Luciano Buttolo, Diego Bertoli, Giorgio Biasiotto, Michele Malagola, Riccardo Marnoni, Mirko Farina, Vera Radici, Simona Bernardi, Alessandro Leoni, Domenico Russo, Daniele Avenoso

**Affiliations:** ^1^ Unit of Blood Diseases and Bone Marrow Transplantation, Department of Clinical and Experimental Science University of Brescia, ASST Spedali Civili di Brescia Brescia Italy; ^2^ Department of Histopathology University of Brescia, ASST Spedali Civili di Brescia Brescia Italy; ^3^ Division of Neurosurgery University of Brescia, ASST Spedali Civili di Brescia Brescia Italy; ^4^ Highly Specialized Laboratory ASST Spedali Civili di Brescia Brescia Italy; ^5^ Department of Molecular and Translational Medicine University of Brescia Brescia Italy

1

Late relapse of acute myeloid leukemia (AML) is the main reason for treatment failure after a successful allogeneic hematopoietic stem cell transplant (allo‐HSCT). A rare manifestation of disease reoccurrence is isolated myeloid sarcoma. A 67‐year‐old gentleman underwent allo‐HSCT in 2020 after achieving second complete remission, following salvage treatment for relapsed AML originally diagnosed in 2018. At the onset, the cytogenetic assay on bone marrow was remarkable for translocation (16;17)(p11;p13); retrospective next‐generation sequencing revealed an acquired mutation in RUNX1, ASXL1, SRSF2, and DNMT3A.

In October 2024, the patient attended our clinic for the onset of a nuchal headache and mild visual disturbances, prompting extensive diagnostic investigations. Magnetic resonance imaging of the central nervous system (CNS) showed the presence of an intra‐axial expansive lesion in the right parietal–temporal lobe (maximum orthogonal diameters were 3.4 × 3.1 cm in T2W—Figure 1A and in T1W sequence—Figure 1B). Histopathological analysis revealed the presence of immature cells (Figure 1C) with partial expression of CD34, MPO, CD117, CD4, and CD163, along with negative staining for CD3, CD20, CD19, and CD1a. Nuclear NPM expression was normal. These findings led to the diagnosis of isolated cerebral myeloid sarcoma. Molecular evaluation of the brain biopsy showed the presence of RUNX1, ASXL1, SRSF2, previously detected at the diagnosis. This finding allowed us to rule out a donor‐derived myeloid neoplasia. Furthermore, the NGS evaluation was negative for any targetable lesion such as FLT3‐ITD, IDH1, or IDH2 but remarkable for new mutations such as STAG2, BCOR, and NRAS, suggesting a clonal evolution of the original disease. Notably, bone marrow biopsy and aspirate confirmed the absence of systemic disease, and chimerism analysis showed 100% donor origin within the CD34+ and CD3 fractions, suggesting that an effective immunological pressure for 4 years was likely the reason for a relapse within a sanctuary organ. Intravenous chemotherapy with cytosine arabinoside (3 g/m^2^) was administered with minimal clinical response. The patient rapidly developed pancytopenia and faced a septic shock due to multi‐susceptible *Pseudomonas aeruginosa* (which prevented an association with radiotherapy). He died after 5 days because of multi‐organ failure.

**FIGURE 1:**
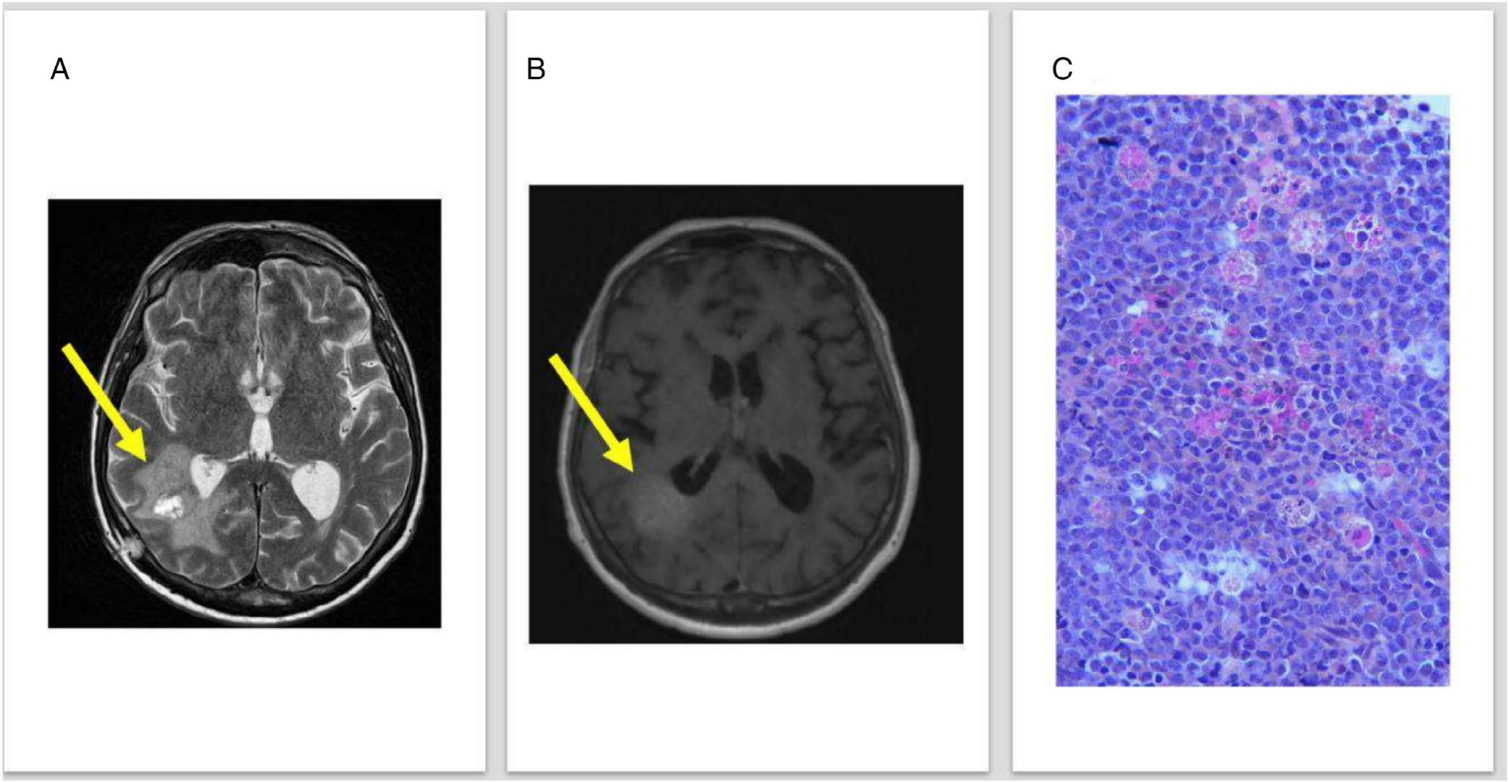
(A) T2W sequence that shows the presence of an intra‐axial expansive lesion in the right parietal–temporal lobe; (B) T1W of the intracerebral neoplastic lesion; (C) Histological section of the tumor showing myeloid blasts admixed with elements of myeloid lineage in various stages of maturation. Numerous mitotic figures are seen. Hematoxylin and eosin, 400× magnification. [Color figure can be viewed at wileyonlinelibrary.com]

This case underscores the critical importance of a multidisciplinary approach in allo‐HSCT recipients, considering the complex interaction between donor immune cells and recipient leukemia cells. It also highlights the necessity of invasive diagnostic procedures for precise disease characterization, which is pivotal for guiding therapeutic strategies and determining prognosis.

## 2 Ethics Statement

Patient provided written consent to collect data for publication. The manuscript was approved by the internal board of the institution.

## Conflicts of Interest

The authors declare no conflicts of interest.

## Data Availability

The authors have nothing to report.

